# Design of a Compact Two-Stage ADC Based on a Nonuniform Flash Architecture for NTC Thermistor Linearization

**DOI:** 10.3390/s26103012

**Published:** 2026-05-10

**Authors:** Jelena Jovanović

**Affiliations:** Faculty of Electronic Engineering, Department of Measurements, University of Niš, Aleksandra Medvedeva 4, 18000 Niš, Serbia; jelena.jovanovic@elfak.ni.ac.rs

**Keywords:** sensor linearization, compact two-stage ADC, nonuniform flash ADC, comparator count, reference resistor ladder, NTC thermistor

## Abstract

This paper proposes a compact two-stage analog-to-digital converter (ADC), leveraging a nonuniform flash ADC to address sensor linearization challenges. The proposed approach reduces circuit complexity in conventional two-stage linearizing ADCs while preserving overall resolution and effective nonlinearity reduction. The compactness of the proposed two-stage ADC is achieved by reducing the number of comparators in the nonuniform flash ADC and reusing the same flash ADC across both conversion stages. Consequently, the total number of comparators is reduced by nearly 75%, while the number of resistors is reduced by 50% compared to a conventional two-stage ADC having the identical resolutions in both stages as the proposed architecture. Nonuniform quantization is implemented using a reference resistor ladder with different resistance values, generating quantization levels that approximate the inverse of the nonlinear input signal and perform linearization. Performance of the proposed two-stage ADC is evaluated through LabVIEW simulations using a Negative Temperature Coefficient (NTC) thermistor (Vishay NTCLE428E3103F400L) as a case study. Results demonstrate a reduction in measurement error from 1 °C to 0.0033 °C in the range from −20 °C to 60 °C, achieving an overall resolution of 20 bits using a 10-bit nonuniform flash ADC.

## 1. Introduction

### 1.1. Sensor Nonlinearity: Problem Definition and Consequences

Sensors are fundamental components in measurement and control systems, converting physical quantities into electrical signals for processing and decision-making. However, the majority of sensors exhibit nonlinear transfer functions due to inherent device physics, transducer mechanics, or interfacing electronics and vary significantly with technology and operating conditions [1]. Sensor nonlinearity manifests as static transfer function deviations from ideal linear response, dynamic amplitude-dependent distortion, and localized inflection behavior across the operating range [2,3].

The consequences of uncorrected sensor nonlinearity are significant. Nonlinear sensor responses reduce measurement accuracy by introducing systematic errors that degrade the fidelity of derived quantities and parameter estimation [1]. In precision applications, even small deviations from linearity can accumulate into unacceptable errors in calculated physical parameters. In control systems, controllers and observers designed under linear assumptions may exhibit gain errors, offset drift, or reduced stability when driven by nonlinear sensor outputs across operating ranges [4]. Since such controllers rely on accurate, preferably linear, state information, uncompensated nonlinearity can lead to offset errors, incorrect gain behavior, or even instability [4]. Moreover, in multi-channel or high-rate systems, real-time compensation imposes computational, memory, and hardware constraints that must be balanced against latency and accuracy requirements [5]. These effects highlight the importance of effective sensor linearization for reliable measurement and control performance.

### 1.2. Overview of Sensor Linearization Techniques

Sensor linearization techniques are generally classified as hardware, software, and hybrid approaches, each reflecting different trade-offs among latency, flexibility, accuracy, power consumption, and implementation complexity.

#### 1.2.1. Hardware Linearization Techniques

Hardware linearization techniques perform correction in the analog or mixed-signal domain before or during analog-to-digital (A/D) conversion, targeting low-latency and resource-constrained deployments. Analog conditioning, such as amplification, gain and offset correction, bridge balancing, and cold-junction compensation, is widely used to reduce sensor nonlinearity before A/D conversion, particularly in thermocouple measurement chains where low-level voltages and junction effects must be handled in analog front-ends [4]. These solutions provide low-latency and reduced digital processing requirements but are less adaptable and require hardware redesign for different sensors [4].

Bridge circuits and matched analog networks are an attractive hardware approach for linearizing or balancing sensor outputs before A/D conversion due to low component count and continuous-time operation, though their correction capability is limited compared to digital methods [1].

A more sophisticated hardware approach involves an analog-to-digital converter (ADC) with a tailored transfer function designed to implement sensor linearization directly in the conversion stage itself. Lopez-Martin et al. proposed a Complementary Metal-Oxide-Semiconductor (CMOS) ADC with a piecewise-linear (PWL) transfer function approximating the inverse sensor response [6], while Volpi et al. presented a nonlinear ADC tailored for sensor linearization [7]. Such architectures enable deterministic, low-latency conversion but are sensor-specific, requiring modifications to voltage thresholds when applied to different sensor types [6,7]. The linearization technique proposed in this study follows a similar hardware-centric principle, utilizing a dedicated ADC transfer function to achieve direct linearization.

#### 1.2.2. Software Linearization Techniques

Software-based linearization, applied after A/D conversion, offers high flexibility and is widely implemented in microcontrollers and data acquisition systems. Polynomial fitting and progressive polynomial calibration represent the most straightforward software approach, mapping nonlinear sensor outputs to physical quantities through global polynomial fits or staged polynomial correction [8,9]. These methods are simple and memory-efficient but may lose accuracy for highly nonlinear characteristics or sensors with inflection points, or introduce numerical instability at higher degrees [8,9].

Lookup tables (LUTs) and PWL approximation methods approximate inverse sensor characteristics with predictable error bounds and low runtime cost, making them suitable for embedded systems [3]. However, they require memory storage for LUTs and segmentation overhead, and the inverse sensor relation must be computed offline [3]. Marinov et al. developed a PWL approximation algorithm for differentiable sensor characteristics with inflection points, specifically designed to minimize memory and computational cost on resource-constrained microcontrollers and applied to K-type thermocouple linearization [3]. The algorithmic assumptions about sensor smoothness and curvature, along with the need for thresholds selection and calibration data, represent the primary limitations [3].

Engin and Engin developed a 32-bit microcontroller system that integrates cold-junction compensation, amplification, A/D conversion, and software linearization using piecewise and polynomial methods for K-type thermocouples [4]. The design offers flexibility and simple calibration, and avoids hardware redesign for new sensors, though it is limited by runtime, memory, and ADC front-end quality [4].

Neural networks and nonlinear multivariate models are software techniques capable of modeling complex sensor responses and cross-sensitivities. Dieterle et al. compared approaches to multivariate calibration of nonlinear sensor data, including neural networks and regression models [10]. These techniques can approximate continuous nonlinear mappings and manage multivariate dependencies, but risk overfitting, demand higher computational resources, and require representative training datasets [10].

Spline interpolation and piecewise polynomial methods provide smooth inverse mappings with local control over approximation error [1]. Compared to simple PWL schemes, they improve smoothness but add computational and storage overhead.

Sonowal and Bhuyan compared piecewise linearization, lookup table, interpolation, and neural network methods for multi-channel thermistor data on a field-programmable gate array (FPGA) [5]. FPGAs can host multiple linearization algorithms for real-time multi-channel acquisition, balancing logic use and accuracy through parallelism, deterministic timing, and reconfigurability [5]. Yet, higher-accuracy methods demand more logic and memory, while fixed-point arithmetic introduces quantization and rounding challenges [5].

#### 1.2.3. Hybrid Approaches

Hybrid approaches combine analog preprocessing with digital compensation, using ADCs with tailored transfer functions supplemented by digital correction to reduce computational load while maintaining adaptability [6,7].

Active digital precompensation is a hybrid linearization approach in which an inverse model of the transducer is applied to the driving signal in order to compensate for nonlinear behavior at the system output [2]. Klippel demonstrated reduced harmonic distortion and extended linear operating range in electroacoustic and electromechanical transducers using this linearization method [2]. However, it requires accurate transducer modeling, increased digital processing effort, and careful management of latency and stability [2].

In embedded systems, analog front-ends for cold-junction compensation, amplification and A/D conversion are frequently combined with polynomial or piecewise digital linearization [4]. This combination leverages the strengths of both domains and enables firmware flexibility but increases cross-domain design and calibration complexity [4].

### 1.3. ADC-Based Sensor Linearization and Flash Architectures

Building upon the hardware linearization approaches discussed in Section 1.2, this section focuses specifically on ADC-based implementations, with particular emphasis on flash architectures relevant to high-speed and low-latency applications. ADC-based linearization techniques, such as those reported by Lopez-Martin et al. [6] and Volpi et al. [7], embed the inverse sensor transfer function directly into the converter transfer function, enabling low-latency operation but requiring sensor-specific threshold design. Similarly, the architecture presented in this study requires specific hardware adjustments of the voltage thresholds to match the nonlinear response of the target sensor.

Flash ADCs provide key advantages for sensor linearization due to their fully parallel, comparator-based structure, enabling extremely low latency and very high conversion speeds suitable for real-time correction in fast sensor systems. Because quantization is completed within one comparator stage, high-speed flash converters achieve multi-GS/s sampling rates, as shown in wideband communication and instrumentation systems [11]. Simultaneous operation of all comparators ensures parallel threshold detection, reducing conversion-time uncertainty and providing deterministic timing critical for consistent per-sample linearization [11]. For instance, Ayesh et al. implemented a time-interleaved charge-steering flash ADC in 20-Gb/s front-end systems, demonstrating real-time feasibility in high-speed environments [12]. However, to fully leverage these high-speed benefits in practical sensor interfaces, one must address the inherent architectural trade-offs, specifically regarding power consumption, silicon area, and resolution.

Despite speed advantages, these significant overheads often limit the feasibility of flash-based linearization. High power consumption arises from the large comparator array and reference resistor ladder, with high-speed designs at tens of GS/s reporting watt-level dissipation [11]. Large silicon area and input capacitance further challenge sensor front-ends, though techniques like interpolation or preamplifier averaging can mitigate these effects [13]. Design complexity also increases due to encoder design, comparator matching and calibration, and reference resistor ladder requirements [14,15]. While optimizations such as encoder refinement or standby comparator schemes reduce power and area, they add further complexity [14,15].

Furthermore, the resolution *N* of a flash ADC is fundamentally constrained: the comparator count (2*^N^* − 1) grows exponentially with *N*, making high-resolution flash ADCs impractical for precision sensor applications. Thus, they are typically limited to low- or moderate-resolution applications unless the hardware complexity is mitigated.

Several strategies address these drawbacks while preserving conversion speed. Comparator power gating and adaptive resolution switching reduce power consumption [15], while charge-steering comparators lower dynamic consumption in very high-speed designs [12]. Encoder optimizations minimize area, delay, and power consumption [14], and interpolation or averaging networks improve linearity while partially mitigating input capacitance issues [13]. For example, Soleimani and Toofan reported encoder improvements that reduced encoder power consumption from 17.94 mW to 11.74 mW in a 5-bit ADC while maintaining a comparable effective number of bits (ENOB) [14]. By implementing such optimizations, it becomes possible to maintain the high-speed effectiveness of flash ADCs for real-time sensor linearization while keeping the hardware footprint within acceptable limits.

### 1.4. Motivation and Proposed Linearization Approach

Although many sensor linearization techniques have been reported, important limitations remain. Hardware-based methods provide very low latency but are generally inflexible; once implemented, they cannot easily adapt to different sensor types or updated calibration requirements without redesigning the circuitry [6,7]. Software-based methods offer high flexibility and easy reconfiguration but require computational resources that may be unavailable in constrained systems or impractical in high-speed applications [3,4,5]. Hybrid approaches attempt to combine hardware and software advantages but demand complex co-design of analog and digital subsystems and careful calibration across domains, increasing system complexity [2,4].

Flash ADCs, despite their inherent speed and low latency, have been underutilized for sensor linearization due to the aforementioned disadvantages in power consumption, silicon area, and limited resolution [11,13]. This research is motivated by the need to bridge this gap [16], retaining the deterministic advantages of flash architectures while overcoming their hardware-intensive nature.

This paper proposes a sensor linearization approach based on a flash ADC, wherein PWL correction is directly integrated into the converter. More specifically, a compact two-stage linearizing ADC based on a nonuniform flash ADC is introduced. The proposed architecture directly addresses the exponential hardware growth of traditional flash ADCs by reducing the comparator count in the nonuniform flash ADC and reusing the same flash ADC across both conversion stages. In this way, the effective resolution of the converter is maintained while the hardware complexity is significantly reduced, effectively mitigating the primary disadvantages that typically hinder flash-based linearization. Nonuniform (i.e., PWL) quantization levels, corresponding to the comparators’ reference voltages, are obtained through a reference resistor ladder composed of resistors of different values. As a result, the proposed linearization approach embeds the inverse function of the input nonlinear signal directly into the flash ADC transfer function, eliminating the need for additional digital post-processing. At the same time, flexibility is retained through adjustable reference voltages, allowing the architecture to be adapted to different sensor types and operating ranges. To be precise, if the sensor characteristics change (i.e., its transfer function is modified, either by using a different sensor model or a different sensor type altogether), the resistor values in the reference ladder can be tuned accordingly. This represents a distinct advantage over other hardware-based solutions, which would require a full architectural redesign to accommodate a new sensor. By contrast, the proposed architecture allows the same hardware platform to be reconfigured by merely adjusting or replacing the resistor values in the reference ladder, thereby streamlining the redesign process and minimizing cost. This targeted hardware-level reconfiguration ensures that the core circuit design remains stable across different applications, prioritized for speed and low latency, while avoiding the full redesign complexity.

To validate the proposed approach, a Negative Temperature Coefficient (NTC) thermistor was selected as a case study. NTC thermistors are widely used temperature sensors characterized by a strongly nonlinear resistance-temperature relationship that follows an exponential behavior. Numerous linearization techniques for NTC thermistors have been reported in the literature, including: hardware-based solutions such as Chebyshev-optimal analog circuits, bridge networks, and serial-parallel resistor configurations [17,18]; software-based methods such as lookup tables, polynomial approximations, the Steinhart-Hart equation, and the B-parameter equation [19,20]; and machine-learning-based techniques, including deep neural networks [21]. Recent comparative analyses have evaluated the trade-offs between accuracy and execution time for different NTC thermistor linearization methods implemented on microcontrollers, providing useful performance benchmarks [19].

The performance of the proposed two-stage linearizing ADC is evaluated through simulations conducted in the LabVIEW 2023 environment [22]. The obtained results confirm that the proposed architecture provides an efficient and compact solution for high-resolution sensor linearization while substantially reducing circuit complexity.

The remainder of this paper is organized as follows. Section 2 presents the system design and methods for NTC thermistor linearization, including interfacing circuits and the development of a compact two-stage linearizing ADC. Section 3 presents the simulation framework and numerical results, highlighting the effectiveness of nonlinearity compensation and the reduction in hardware complexity, and discusses the main findings along with potential directions for future research and design optimization. Finally, Section 4 summarizes the main contributions of this study.

## 2. System Design and Methods

### 2.1. NTC Thermistors

Negative Temperature Coefficient (NTC) thermistors are semiconductor resistive temperature sensors whose resistance exhibits a pronounced nonlinear dependence on temperature. They are widely exploited for their high measurement accuracy, reliability, fast thermal response, and compact dimensions. Their defining property is an exponential decrease in resistance as temperature increases, meaning that they possess a negative temperature coefficient. At room temperature, their nominal resistance can range from several kilo-ohms up to approximately 40 MΩ [23]. In practical applications, NTC thermistors are typically employed within a temperature interval from −50 °C to 200 °C [23].

Compared with metallic resistance temperature detectors (RTDs), NTC thermistors offer several advantages, including smaller physical size, greater temperature sensitivity, and higher nominal resistance values, which reduce the influence of lead-wire resistance on measurement accuracy. For instance, the temperature sensitivity of an NTC thermistor is roughly an order of magnitude higher than that of a platinum-based resistive thermometer [24]. Nevertheless, this sensitivity is not constant; it progressively decreases as temperature rises. Consequently, NTC thermistors are generally preferred in applications involving relatively narrow temperature spans, such as medical diagnostics, biological systems, meteorological monitoring, and heating, ventilation, and air conditioning (HVAC) systems.

**Figure 1:**
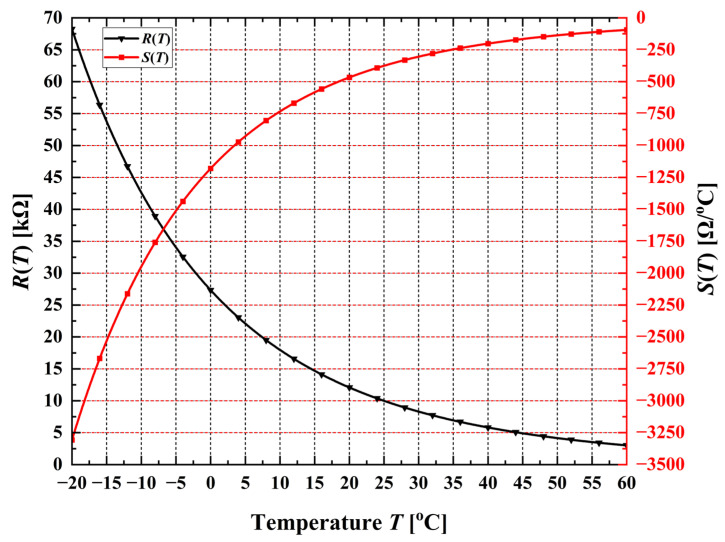
Temperature dependence of resistance *R*(*T*) and sensitivity *S*(*T*) for the Vishay NTCLE428E3103F400L thermistor.

The static resistance-temperature characteristic *R*(*T*) of an NTC thermistor is strongly nonlinear, as illustrated in Figure 1. The figure shows both the static transfer function *R*(*T*) and the corresponding sensitivity *S*(*T*) for the Vishay NTCLE428E3103F400L thermistor [25], evaluated over the temperature range from −20 °C to 60 °C.

The static characteristic *R*(*T*) of this thermistor can be accurately described using the Steinhart-Hart model [26], expressed as follows:

(1) $$R\left(T\right)=R_{25\;{}^{\circ}C}\cdot \exp\left(A+\frac{B}{T}+\frac{C}{T^{2}}+\frac{D}{T^{3}}\right),$$

where *T* denotes the absolute temperature in kelvins [K], *R*_25°C_ = 10 kΩ represents the nominal resistance at 25 °C, and A = −12.89228328, B = 4245.148, C = −87493, and D = −9588114 are the Steinhart-Hart coefficients corresponding to the Vishay NTCLE428E3103F400L thermistor [25].

The temperature dependence of the sensitivity is given by the following:

(2) $$S\left(T\right)=\frac{dR\left(T\right)}{dT}=-R\left(T\right)\cdot \left(\frac{B}{T^{2}}+\frac{2\cdot C}{T^{3}}+\frac{3\cdot D}{T^{4}}\right),$$

where *T* is expressed in kelvins [K], and A, B, C, and D are the same Steinhart-Hart coefficients. Owing to the inherent nonlinearity of the resistance-temperature characteristic, the sensitivity *S*(*T*) varies across the measurement range; specifically, its absolute value decreases with increasing temperature [23]. The objective of linearization is therefore to convert the nonlinear transfer characteristic, i.e., the dependence of the measured electrical quantity on the input physical parameter, into an approximately linear relationship while maintaining a nearly constant sensitivity over the entire operating range.

### 2.2. NTC Thermistor Linearization Circuits

NTC thermistor-based temperature measurement circuits, commonly called thermistor thermometers or interfacing circuits, are typically realized as a resistive voltage divider [27,28,29] or a Wheatstone bridge [23,27,28,30,31]. Appropriate selection of component values allows partial hardware compensation of thermistor nonlinearity, but only over limited temperature ranges. Consequently, the output voltage is pseudo-linear rather than strictly linear [24,27,29], requiring additional linearization to achieve higher measurement accuracy.

Hardware (analog) linearization solutions include circuits based on the 7555 timer [32], combinations of logarithmic, differential, and analog divider stages [33], and op-amp inverting amplifier configurations [34]. Mixed-signal hardware linearization is commonly implemented using ADCs [6,7,29,31,35,36,37,38,39,40,41]. In addition to these approaches, software methods, such as lookup tables, are used [5].

Various ADC architectures have been proposed for linearizing different sensor types, including NTC thermistors [29,31,37,38,39,40,41]. In [38], an NTC thermistor and a fixed resistor generate input currents for a logarithmic amplifier whose output is processed by a linearizing dual-slope ADC, achieving ±0.2% residual nonlinearity. In [31], a dual-slope ADC linearizes a Wheatstone bridge output in quarter-bridge (one resistive sensor) and half-bridge (two resistive sensors) configurations; in the quarter-bridge case, the worst-case residual nonlinearity was ±0.14%. However, dual-slope ADCs inherently involve a compromise between low power consumption and reduced sampling rate.

Most linearizing ADCs employ a two-stage architecture: the first stage performs PWL approximation of the inverse sensor output signal, while the second stage reduces the quantization error introduced by the first stage. There are implementations where in both stages flash ADCs are employed [29]. An alternative approach uses a flash ADC in the first stage and a successive approximation (SAR) ADC in the second stage [41]. The flash-SAR combination reduces power consumption compared to double-flash solutions, though at the cost of increased processing time.

### 2.3. NTC Thermistor Interfacing Circuit

This study addresses the linearization of the NTC thermistor Vishay NTCLE428E3103F400L [25]. The considered linearization range spans from −20 °C to 60 °C. The proposed solution combines a Wheatstone bridge front-end and instrumentation amplifier with a compact two-stage linearizing ADC employing a nonuniform flash ADC.

**Figure 2:**
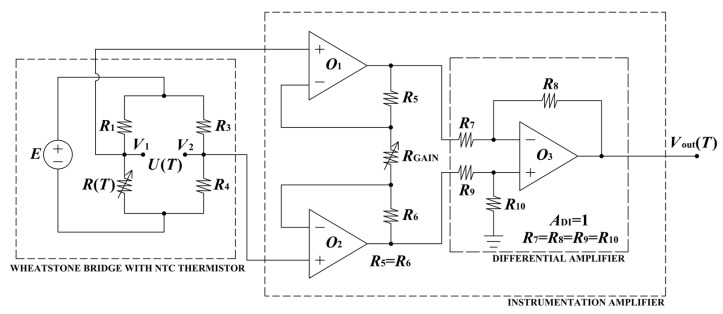
An interfacing circuit for the NTC thermistor, with dashed lines indicating the Wheatstone bridge, the instrumentation amplifier and its internal differential amplifier stage.

The differential bridge output voltage, *U*(*T*) = *V*_1_ − *V*_2_, remains nonlinear and assumes negative values within the selected temperature range. For this reason, before being applied to the linearizing ADC, the bridge output is processed by an instrumentation amplifier (see Figure 2).

The output signal of the Wheatstone bridge is defined as follows:

(3) $$U\left(T\right)=E\cdot \left(\frac{R\left(T\right)}{R_{1}+R\left(T\right)}-\frac{R_{4}}{R_{3}+R_{4}}\right).$$

This signal is *S*-shaped with a single inflection point [27,29]. The temperature corresponding to this inflection point can be deliberately selected, enabling controlled reshaping of the voltage-temperature relationship. It should be emphasized that the highest degree of linearity of *U*(*T*) occurs in the vicinity of the inflection point. Therefore, the location of the inflection point sets the temperature subrange that exhibits the highest linearity. In this study, the inflection point is chosen to be at 20 °C.

#### 2.3.1. Wheatstone Bridge-Based Thermistor Front-End

Determination of the Wheatstone bridge parameters *R*_1_, *R*_3_, *R*_4_, and the supply voltage *E* requires the following design steps:

1. Maximum permissible thermistor current *I*_p_.

The maximum permissible current through the NTC thermistor must not produce a self-heating error exceeding Δ*T* = 0.05 °C. The corresponding condition is given by the following:

(4) $$I_{p}=\sqrt{\frac{\delta \cdot \Delta T}{R{\left(T\right)}_{\min}}}=0.418\;\mathrm{mA},$$

where, for the particular thermistor, the dissipation constant is *δ* = 3 mW/°C, and the minimum thermistor resistance is *R*(*T*)_min_ = *R*(*T*_max_) = *R*(105 °C) = 858.33 Ω [25]. This resistance corresponds to the highest temperature within the measurement range (the thermistor’s full rated range extends from −40 °C to 105 °C).

2. Determination of the resistance *R*_1_.

The value of the resistor *R*_1_ is calculated from the Wheatstone bridge linearization criterion, defined by the second derivative condition: *U*″(*T*_ip_) = 0 [24]. From this condition, and for the inflection point temperature *T*_ip_ specified in [K], the value of the resistor *R*_1_ can be calculated using the following expression:

(5) $$R_{1}\left(T_{\mathrm{ip}}\right)=R\left(T_{\mathrm{ip}}\right)\cdot \frac{{\left[g^{\prime }\left(T_{\mathrm{ip}}\right)\right]}^{2}-g^{\prime \prime }\left(T_{\mathrm{ip}}\right)}{{\left[g^{\prime }\left(T_{\mathrm{ip}}\right)\right]}^{2}+g^{\prime \prime }\left(T_{\mathrm{ip}}\right)},$$

where the functions *gʹ*(*T*_ip_) and *gʺ*(*T*_ip_) are given by the following:

(6) $$g^{\prime }\left(T_{\mathrm{ip}}\right)=-\left(\frac{B}{T_{\mathrm{ip}}^{2}}+\frac{2\cdot C}{T_{\mathrm{ip}}^{3}}+\frac{3\cdot D}{T_{\mathrm{ip}}^{4}}\right),$$

(7) $$g^{\prime \prime }\left(T_{\mathrm{ip}}\right)=\frac{2\cdot B}{T_{\mathrm{ip}}^{3}}+\frac{6\cdot C}{T_{\mathrm{ip}}^{4}}+\frac{12\cdot D}{T_{\mathrm{ip}}^{5}}.$$

The general expression for the function *g*(*T*) is as follows:

(8) $$g\left(T\right)=A+\frac{B}{T}+\frac{C}{T^{2}}+\frac{D}{T^{3}},$$

when the thermistor behavior is described by the Steinhart-Hart model in the following form:

(9) $$R\left(T\right)=R_{25{}^{\circ}C}\cdot \exp\left(g\left(T\right)\right).$$

For the selected inflection point temperature *T*_ip_ = 20 °C (293.15 K) (which lies midway between *T*_L_ = −20 °C with *R*(*T*_L_) = 68259.59 Ω and *T*_H_ = 60 °C with *R*(*T*_H_) = 3019.66 Ω), the resistance *R*_1_ is calculated as *R*_1_(20 °C) = 9065.77 Ω. The adopted standard value is *R*_1_(20 °C) = 9090 Ω selected from the E192 series of preferred resistances (with a tolerance of 0.5%) as defined by IEC 60063:2015 [42].

3. Determination of the supply voltage *E*.

From the circuit in Figure 2, the bridge excitation voltage *E* can be calculated as follows:

(10) $$E=I_{p}\cdot \left(R_{1}+R{\left(T\right)}_{\min}\right)=4.16\;V.$$

4. Determination of resistances *R*_3_ and *R*_4_.

The remaining bridge resistors are obtained from the balancing condition imposed at the lower boundary of the operating range, *T*_L_ = −20 °C [30]. The bridge is balanced when

(11) $$U\left(T_{L}\right)=E\cdot \left(\frac{R\left(T_{L}\right)}{R_{1}+R\left(T_{L}\right)}-\frac{R_{4}}{R_{3}+R_{4}}\right)=0\;V.$$

This requirement leads to the following relationship:

(12) $$\frac{R_{3}}{R_{4}}=\frac{R_{1}}{R\left(T_{L}\right)}.$$

The standard resistance values for *R*_3_ and *R*_4_, which satisfy the previously defined ratio, are 1060 Ω and 7960 Ω, respectively. These values are also taken from the E192 series in accordance with the IEC 60063:2015 standard [42].

#### 2.3.2. Instrumentation Amplifier

The output voltage of the instrumentation amplifier is defined by the following expression:

(13) $$V_{\mathrm{out}}\left(T\right)=\frac{R_{8}}{R_{7}}\cdot \left(\frac{R_{5}+R_{6}}{R_{\mathrm{GAIN}}}+1\right)\cdot \left(V_{2}-V_{1}\right),$$

which shows that the overall amplification is determined by two factors: the resistor ratio *R*_8_/*R*_7_ in the differential stage and the gain-setting term ((*R*_5_ + *R*_6_)/*R*_GAIN_ + 1) in the input stage (see Figure 2). The output voltage is therefore directly proportional to the differential input signal (*V*_2_ − *V*_1_), while the gain magnitude is controlled by the selected resistor values. For proper differential operation and symmetrical signal processing, Equation (13) holds under the condition that the resistor pairs in the output stage are matched, specifically *R*_7_ = *R*_9_ and *R*_8_ = *R*_10_. This matching ensures equal amplification of both input branches and preserves balanced operation of the amplifier, thereby improving its common-mode rejection. If the differential-stage gain is chosen to be unity, i.e.,

(14) $$A_{\mathrm{DI}}=\frac{R_{8}}{R_{7}}=1,$$

then *R*_8_ = *R*_7_, which consequently implies *R*_7_ = *R*_8_ = *R*_9_ = *R*_10_. Under these conditions, the gain contribution of the differential stage becomes equal to one, and the total amplification is governed solely by the input-stage network. When the condition *R*_5_ = *R*_6_ holds, the output voltage expression simplifies to the following:

(15) $$V_{\mathrm{out}}\left(T\right)=\left(\frac{2\cdot R_{5}}{R_{\mathrm{GAIN}}}+1\right)\cdot \left(V_{2}-V_{1}\right)=A_{T}\cdot \left(V_{2}-V_{1}\right),$$

so that the total voltage gain of the instrumentation amplifier can be written as follows:

(16) $$A_{T}=\frac{2\cdot R_{5}}{R_{\mathrm{GAIN}}}+1.$$

When the resistor value *R*_5_ is known and fixed, the overall gain is adjusted exclusively by the external resistor *R*_GAIN_, while the internal resistor symmetry remains unchanged. This simplifies the practical design, since the desired amplification factor can be set by selecting a single external component without affecting the internal matching of the amplifier. The total gain of the instrumentation amplifier *A*_T_ is determined from the requirement that the maximum input voltage of the two-stage linearizing ADC reaches 5 V at the highest considered temperature, *T*_H_ = 60 °C. In other words, the gain *A*_T_ is selected so that the amplified voltage from the bridge output fully utilizes the input dynamic range of the linearizing ADC at the higher limit of the considered temperature range.

Accordingly, *A*_T_ is defined as the ratio between the linearizing ADC full-scale input voltage (5 V) and the absolute maximum value of the Wheatstone bridge output voltage, |*U*(*T*)_max_|, which occurs at *T*_H_ = 60 °C. This approach ensures optimal scaling of the signal prior to A/D conversion, maximizing resolution while preventing saturation of the ADC input stage. Therefore,

(17) $$A_{T}=\frac{2\cdot R_{5}}{R_{\mathrm{GAIN}}}+1=\frac{5\;V}{\left|U{\left(T\right)}_{\max}\right|}=\frac{5\;V}{\left|U\left(T_{H}\right)\right|}=\frac{5\;V}{2.634\;V}=1.898.$$

When the instrumentation amplifier is implemented using integrated devices, such as INA118 manufactured by Texas Instruments [43], the internal resistor values are fixed as *R*_5_ = *R*_6_ = 25 kΩ and *R*_7_ = *R*_8_ = *R*_9_ = *R*_10_ = 40 kΩ. Consequently, the required gain is obtained by selecting an appropriate value of *R*_GAIN_. In this study, and for the INA118 amplifier, the external resistor was chosen as *R*_GAIN_ = 28  kΩ (from the E192 series in accordance with the IEC 60063:2015 standard [25]), which provides the amplification factor of 1.898 necessary for the subsequent signal processing stage.

#### 2.3.3. Nonlinearity of the Output Voltage V_out_(T)

Nonlinearity error is typically defined for sensors whose transfer function can be approximated by a linear function of the form *y* = a + b·*x* [44]. Whenever a nonlinearity value is reported, it is essential to clearly indicate the reference linear function with respect to which the deviation has been calculated. One common approach for defining the approximation line is the endpoint method. In this approach, a reference straight line is constructed by connecting the sensor’s output values at the minimum and maximum boundaries of the input range.

**Figure 3:**
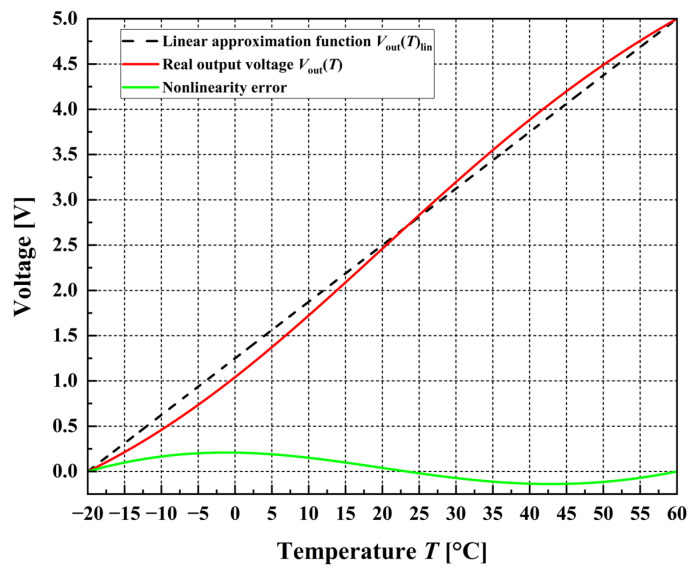
Nonlinear (real) output voltage *V*_out_(*T*), its linear approximation *V*_out_(*T*)_lin_ and the nonlinearity error in the observed temperature range.

In this study, the nonlinearity is defined as the maximum deviation between the output voltage *V*_out_(*T*) and its linear approximation [45], where the coordinates of the starting and ending points are identical for both the nonlinear voltage function and its linear approximation. In other words, the linear approximation passes exactly through the endpoints of the nonlinear voltage function over the considered temperature range. The corresponding linear approximation function is given by the following:

(18) $$V_{\mathrm{out}}{\left(T\right)}_{\mathrm{lin}}=0.0625\cdot T+1.25,$$

where temperature is expressed in [°C] and the output voltage in [V]. As illustrated in Figure 3, this approach results in the smallest nonlinearity error in the vicinity of the endpoints and also near the inflection point, as expected.

In practical measurement scenarios, a particular range of the full measurement interval may be more critical than the rest. In such cases, it is advantageous to minimize the nonlinearity error specifically within that range of interest. This objective can be accomplished by positioning the inflection point at the center of the selected range, thereby reducing the local deviation from linear behavior. In this study, the inflection point is also set in the middle of the observed range, i.e., at 20 °C.

Furthermore, the relative nonlinearity error, denoted as *δ*_NL_, is defined as follows:

(19) $$\delta_{\mathrm{NL}}=\frac{\Delta V_{\max}}{FS}\cdot 100\%,$$

where Δ*V*_max_ [V] denotes the maximum absolute difference between the nonlinear output signal *V*_out_(*T*) and its linear approximation *V*_out_(*T*)_lin_ (see Figure 3), while the *FS* [V] represents the width of the full-scale range, in this case equal to the maximum value of *V*_out_(*T*) [39,44]. The calculated relative nonlinearity error equals to 4.18% and will be further reduced using the proposed two-stage linearizing ADC of a compact design.

### 2.4. Design Principles of a Two-Stage Linearizing ADC Based on a Nonuniform Flash Architecture

A defining characteristic of a two-stage linearizing ADC proposed in this paper is that both conversion stages are performed by a single nonuniform flash ADC. The corresponding nonuniform transfer (quantization) function of the employed flash ADC is composed of multiple linear segments with unequal widths, which are separated by threshold values commonly referred to as voltage thresholds (breakpoints) [40]. These segments approximate the ideal transfer function of the flash ADC, which corresponds to the inverse function of the input signal, i.e., the inverse function of the sensor-interface output voltage *V*_out_(*T*). The second conversion stage is performed by the same nonuniform flash ADC already used in the first stage.

**Figure 4:**
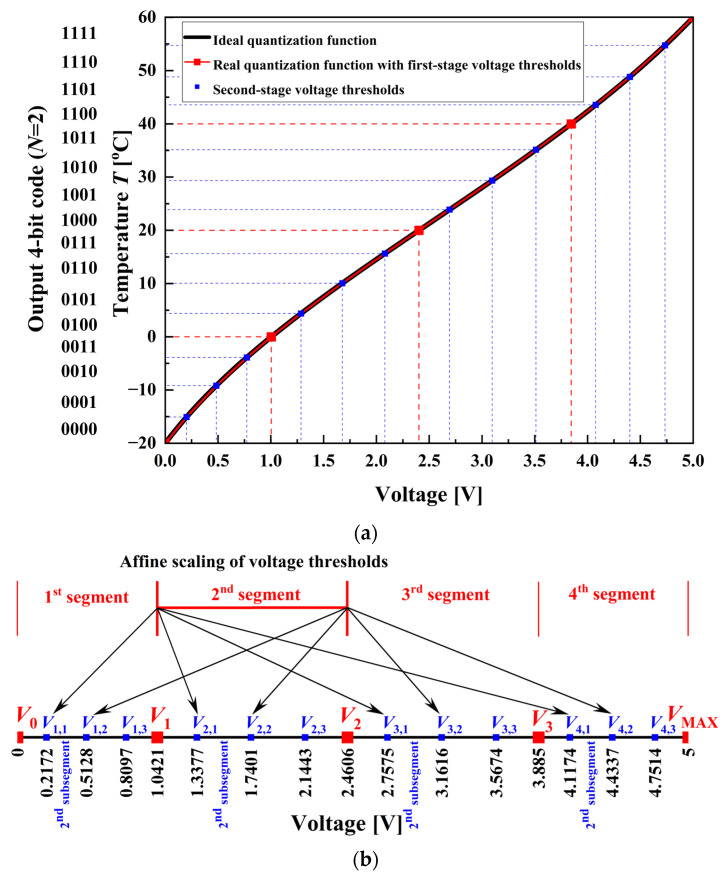
Quantization behavior and affine scaling principle in the proposed 4-bit two-stage linearizing ADC: (**a**) Ideal quantization function (black curve) and the corresponding real quantization function (red lines), showing the first-stage nonuniform voltage thresholds (red squares) and the second-stage nonuniform voltage thresholds (blue squares). (**b**) Illustration of affine scaling of voltage thresholds: second-stage voltage thresholds (blue squares) are obtained by scaling and shifting the first-stage voltage thresholds (red squares) within each segment (arrows exemplify the scaling and shifting of the 2nd segment voltage thresholds).

Figure 4a illustrates the ideal monotonically rising quantization function of a 4-bit two-stage linearizing ADC (continuous black curve). This characteristic is approximated by several linear segments of different widths (red lines), whose endpoints correspond to nonuniformly spaced voltage thresholds (red squares). In the example considered here, each stage of the conversion process has a resolution of *N* = 2 bits. Consequently, the overall quantization function consists of four nonuniform segments, where each segment is further divided into four nonuniform subsegments. This relatively small resolution is chosen solely for the purpose of simplifying the explanation of the design principle. Figure 4b further clarifies the relationship between the two conversion stages by illustrating the affine scaling of voltage thresholds. Once a nonuniform segment is selected in the first stage, the second-stage voltage thresholds are generated by scaling and shifting the same reference structure within the corresponding segment. In this way, the relative spacing of the voltage levels is preserved, while the thresholds are adapted to the local interval, enabling consistent quantization behavior across all segments without requiring an additional reference resistor ladder. In Figure 4b, the second segment is selected as an illustrative example and is scaled and shifted to generate the corresponding second subsegment within each segment.

The first-stage voltage thresholds are determined by first dividing the observed temperature range from *T*_L_ = −20 °C to *T*_H_ = 60 °C (shown on the *y*-axis of Figure 4a) into 2*^N^* segments of equal width. The endpoints of these segments are then mapped onto the voltage axis (*x*-axis of Figure 4a). In general, the first-stage voltage thresholds can be obtained using the following relation:

(20) $$V_{k}=V_{\mathrm{out}}\left(T_{k}\right),\;T_{k}=T_{L}+k\cdot \frac{T_{H}-T_{L}}{2^{N}},\;k=1,2,\ldots ,2^{N}-1,$$

where *k* represents the ordinal number of the first-stage voltage threshold, *N* is the resolution of the flash ADC, and *T_k_* is the corresponding temperature threshold. It is also important to note that voltages *V*_0_ = 0 V (for *T*_L_ = −20 °C) and *V*_MAX_ = 5 V (for *T*_H_ = 60 °C) do not represent reference voltages of the comparators in the flash ADC; however, they determine the input range of the flash ADC in the first conversion stage.

As an illustration, the third segment lies between the voltage thresholds *V*_2_ and *V*_3_. Within this interval, nonuniform subsegments are distributed. The second-stage voltage thresholds are denoted by *V_i_*_,*j*_; for example, *V*_3,1_, *V*_3,2_, and *V*_3,3_ represent the voltage thresholds within the third segment (see Figure 4b). In this notation, *i* identifies the segment index, while *j* indicates the ordinal number of the voltage threshold inside the *i*-th segment. The second-stage voltage thresholds *V_i_*_,*j*_ are calculated as follows:

(21) $$V_{i,j}=V_{\mathrm{out}}\left(T_{i,j}\right),\;T_{i,j}=T_{iL}+j\cdot \frac{T_{iH}-T_{iL}}{2^{N}},\;i=1,2,\ldots ,2^{N},\;j=1,2,\ldots ,2^{N}-1,$$

where *T_i,j_* is the corresponding temperature threshold, and *T_i_*_L_ and *T_i_*_H_ are the boundaries of the *i*-th temperature subrange corresponding to the *i*-th voltage segment. The widths of the subsegments vary within a given segment due to the use of a nonuniform flash ADC. Moreover, subsegments with the same ordinal index differ across segments, since the segments themselves have unequal widths, which is also a consequence of employing a nonuniform ADC in the first stage of conversion.

The distinction between the first and the second conversion stage is defined by the input-range boundaries of the nonuniform flash ADC. In the second stage, the input range of the flash ADC is determined by the boundaries of the segment determined in the first stage of conversion. Finally, at the end of the second conversion stage, the input voltage sample is assigned to the corresponding subsegment, and the result is expressed as the appropriate 4-bit digital output code (shown along the *y*-axis in Figure 4a).

An important advantage of this design approach for the two-stage linearizing ADC is its low implementation complexity, since it does not require the use of a microcontroller or any other digital processing unit for the determination of segments’ and subsegments’ voltage thresholds.

### 2.5. Operating Principles of the Proposed Two-Stage Linearizing ADC

It is well known that a conventional *N*-bit flash ADC requires 2*^N^*− 1 comparators and 2*^N^* resistors. Consequently, a two-stage architecture that employs two flash ADCs with different resolutions requires 2*^N^*^1^ − 1 + 2*^N^*^2^ − 1 comparators and 2*^N^*^1^ + 2*^N^*^2^ resistors, where *N*_1_ and *N*_2_ denote the resolutions of the first and second conversion stages, respectively. In the proposed two-stage linearizing ADC, both conversion stages are operated using the same nonuniform flash ADC with the resolution *N*, yielding an overall resolution of 2·*N*. In the proposed design, the number of required comparators is 2*^N^*^−1^ (due to the compact design of the flash ADC), while the number of employed resistors is 2*^N^*. This approach leads to a more compact two-stage ADC structure with reduced power consumption.

**Figure 5:**
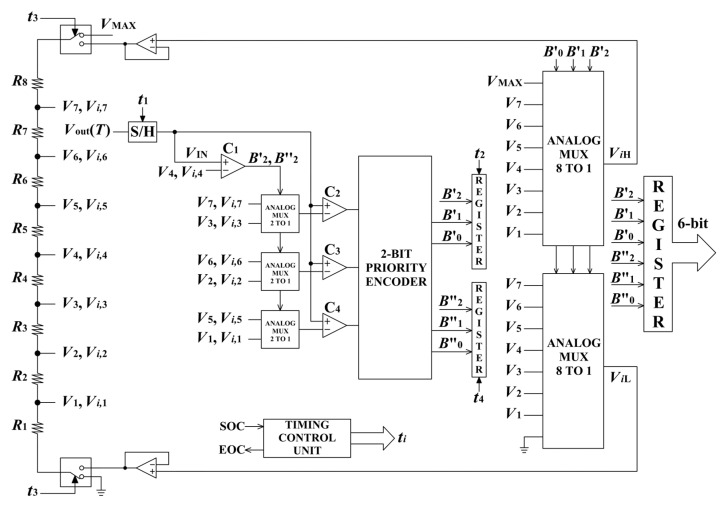
Proposed 6-bit compact two-stage linearizing ADC based on a 3-bit nonuniform flash ADC.

To illustrate the operating principle, a 6-bit two-stage linearizing ADC is considered (see Figure 5). In this configuration, a 3-bit nonuniform flash ADC (*N* = 3) is used. The number of first-stage voltage thresholds is 7, denoted as *V*_1_ to *V*_7_. These voltages are generated using a reference resistor ladder composed of resistors *R*_1_ to *R*_8_, whose resistance values are mutually different. First-stage voltage thresholds are obtained from the reference voltage, which corresponds to the maximum ADC input voltage *V*_MAX_ = (*V*_out_(*T*))_max_ = 5 V. The resistors’ values used to set these voltage thresholds are as follows:

(22) $$R_{i}=\frac{R_{\Sigma }}{V_{\mathrm{MAX}}}\left[V_{\mathrm{out}}\left(T_{i}\right)-V_{\mathrm{out}}\left(T_{i-1}\right)\right],\;i=1,2,\ldots ,2^{N},$$

where *R*_Σ_ represents the sum of the resistances composing the reference resistor ladder, while *V*_out_(*T*_0_) = *V*_out_(*T*_L_) = 0 V and *V*_out_(*T*_2^*N*^_) = *V*_out_(*T*_H_) = 5 V. In the proposed architecture, the parameter *R*_Σ_ is set to 10 kΩ.

The entire conversion procedure is coordinated by timing signals *t_i_* (*i* = 1, 2, 3, 4), which are generated by a timing control unit (see Figure 5). The conversion and linearization processes begin with sampling the input voltage *V*_out_(*T*) (triggered by signal *t*_1_). The sampled value *V*_IN_ is then applied to comparator C_1_, which determines whether the input sample lies below or above the central voltage threshold *V*_4_. The output of comparator C_1_ is the bit *B*′_2_, which represents the most significant bit (MSB) of the final digital result. If *B*′_2_ = 1, the input sample exceeds *V*_4_; otherwise, if *B*′_2_ = 0, the sample lies below this threshold. This bit controls three 2-to-1 analog multiplexers (AMUXs) whose outputs are the voltage thresholds *V*_7_, *V*_6_ and *V*_5_ if *B*′_2_ = 1, or *V*_3_, *V*_2_ and *V*_1_ if *B*′_2_ = 0. The outputs of 2-to-1 AMUXs serve as reference voltages for comparators C_2_, C_3_ and C_4_.

The outputs of these comparators form a thermometer code [46,47,48], which is subsequently converted into two bits, *B*′_1_ and *B*′_0_, using a 2-bit priority encoder. The first three bits *B*′_2_, *B*′_1_ and *B*′_0_ are then stored in the register under the control of the timing signal *t*_2_. These three output bits obtained in the first conversion stage determine the index *i* of the nonuniform segment to which the current input sample *V*_IN_ belongs. In addition, these bits control two 8-to-1 AMUXs, whose outputs define the lower and higher boundary of that segment, denoted *V_i_*_L_ and *V_i_*_H_. These voltages simultaneously define the input range of the second conversion stage.

The second-stage conversion proceeds in a manner analogous to the first stage, except that the operation is now confined within the boundaries of the identified *i*-th segment. Timing signal *t*_3_ activates the switches that select the appropriate input range boundaries for the second stage (*V_i_*_L_ and *V_i_*_H_, instead of 0 V and *V*_MAX_). The reference resistor ladder now generates the subsegments’ boundaries *V_i_*_,*j*_ (*i* = 1, …, 2*^N^*;  *j* = 1, …, 2*^N^*−1; *N* = 3). In contrast to the conventional two-stage ADC, the reference resistor ladder used in the second stage is the same as in the first conversion stage. At this point, the exact nonuniform subsegment containing the current sample is determined and encoded using three additional bits, *B*′′_2_, *B*′′_1_ and *B*′′_0_. After these bits are written into the register (under control of timing signal *t*_4_), the final 6-bit digital output code is obtained. It should be noted that when the complete 6-bit digital output *B*′_2_*B*′_1_*B*′_0_*B*′′_2_*B*′′_1_*B*′′_0_ is converted back into its corresponding analog voltage, and subsequently into the associated temperature value, the difference between the real temperature and the measured value becomes significantly smaller. In other words, the proposed two-stage linearizing ADC significantly reduces the measurement error caused by the inherent nonlinearity of the considered thermistor, as will be demonstrated by the numerical results presented in the following section.

## 3. Results and Discussions

To evaluate the advantages of the proposed two-stage linearizing ADC, its performance is compared with that of a conventional two-stage linearizing ADC. The proposed architecture employs a single *N*-bit nonuniform flash ADC, while the conventional one consists of an *N*_1_-bit nonuniform flash ADC followed by an *N*_2_-bit uniform flash ADC. In both cases, the total resolution is identical and defined by *n* = 2 · *N* = *N*_1_ + *N*_2_. The operating principles of both architectures are analyzed together with their ability to compensate for the nonlinear behavior of the output voltage *V*_out_(*T*) generated by the sensor interface circuit incorporating the Vishay NTCLE428E3103F400L thermistor. The analysis is performed using simulations implemented in the LabVIEW 2023 environment [22]. Both ADC architectures are modeled using dedicated virtual instruments.

**Figure 6:**
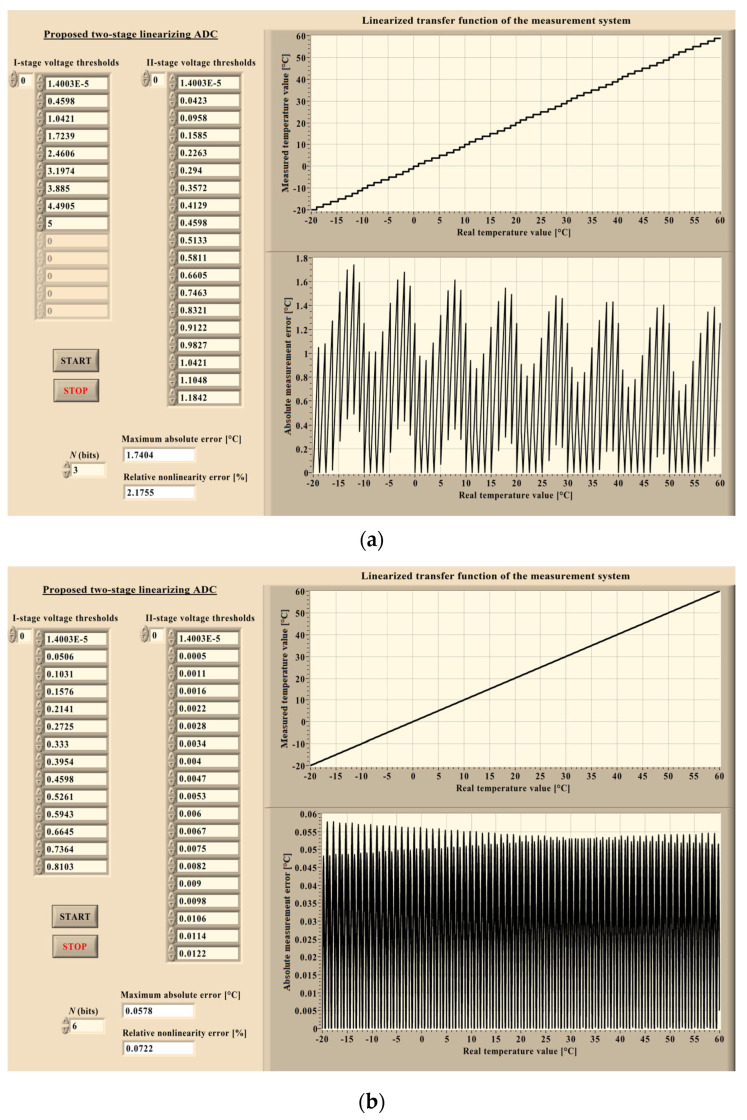
Front panel of the virtual instrument simulating the measurement system based on the proposed two-stage linearizing ADC: (**a**) The case when *N* = 3 bits. (**b**) The case when *N* = 6 bits.

**Figure 7:**
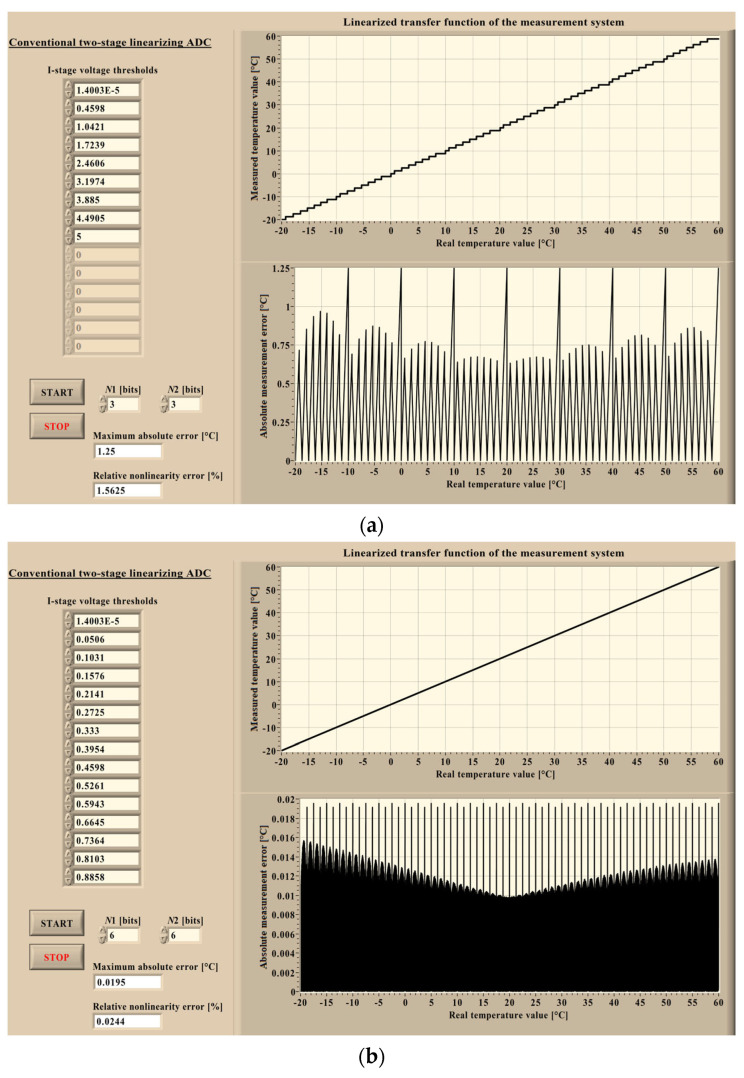
Front panel of the virtual instrument simulating the measurement system based on the conventional two-stage linearizing ADC: (**a**) The case when *N*_1_ = *N*_2_ = 3 bits. (**b**) The case when *N*_1_ = *N*_2_ = 6 bits.

Figure 6 presents the front panel of the virtual instrument developed for the simulation of the complete measurement chain, beginning with the thermistor input and ending with the digital output of the proposed two-stage linearizing ADC. After the conversion process, the obtained digital output is reconverted into the corresponding temperature value to evaluate the measurement accuracy. In Figure 6a, the resolution of the non-uniform flash ADC is set to *N* = 3 bits, whereas in Figure 6b, the resolution is *N* = 6 bits. The results for the conventional architecture are obtained using the virtual instrument illustrated in Figure 7, where case (a) corresponds to ADC resolutions *N*_1_ = *N*_2_ = 3 bits, and case (b) corresponds to *N*_1_ = *N*_2_ = 6 bits.

At the beginning of the simulation procedure, the resolution of the proposed ADC architecture is selected, after which the START command initiates each simulation. In the first step, the voltage thresholds corresponding to the first conversion stage are calculated. These thresholds are then compared with the input voltage sample. Based on these comparisons, the nonuniform segment containing the input sample is identified, and its digital representation is generated. Once the corresponding segment is determined, its lower and higher voltage thresholds are obtained, which define the input range for the second conversion stage. The second conversion stage subsequently determines the ordinal index of the nonuniform subsegment within the selected segment that contains the input sample. This index is then encoded to produce the final digital representation of the input sample. The voltage thresholds associated with the second conversion stage are also displayed on the front panel of the virtual instrument (not all of them are visible due to a great number), simulating the proposed architecture.

The described procedure is repeated for each sample of the input voltage *V*_out_(*T*). After the conversion process is completed, the resulting digital codes are transformed back into temperature values and compared with the corresponding real (input) temperature values. The difference between these quantities represents the measurement error. In this way, the linearized transfer function of the complete measurement system is obtained. In addition, the absolute measurement error is plotted as a function of the real temperature across the considered range. The maximum absolute error and the relative nonlinearity error are also calculated.

As shown in Figure 7, the resolutions of the first and second conversion stages can be selected independently in the conventional architecture. Consequently, the first-stage and second-stage resolutions are not necessarily equal, unlike in the proposed design. In this virtual instrument, the voltage thresholds of the first conversion stage are displayed on the front panel. The thresholds corresponding to the second-stage subsegments are not explicitly listed, as these subsegments have equal widths (uniform spacing), and their thresholds can therefore be directly derived from the first-stage thresholds. The front panel also presents the linearized transfer function of the measurement system together with the absolute measurement error plotted as a function of the real (input) temperature over the considered range. The maximum absolute error and the relative nonlinearity error are calculated in this case as well. These results are summarized in Table 1 for different values of the total resolution *n* (where *n* = 2 · *N* = *N*_1_ + *N*_2_, and *N* = *N*_1_ = *N*_2_).

In Table 1, (Δ*T*_max_)_p_ and (*δ*_NL_)_p_ denote the performance metrics of the proposed architecture. Specifically, (Δ*T*_max_)_p_ represents the maximum absolute error, defined as the maximum absolute difference between the real and measured temperature, while (*δ*_NL_)_p_ denotes the relative nonlinearity error, defined as the ratio of this maximum absolute error to the full-scale range (*FS* = 80 °C, spanning from −20 °C to 60 °C). Correspondingly, (Δ*T*_max_)_c_ and (*δ*_NL_)_c_ represent the values obtained for the conventional architecture.

Both simulations were performed for total ADC resolutions ranging from 6 to 20 bits; however, as the total resolution increases, simulations require substantially more computational resources and time, while the resulting conclusions remain unchanged. The numerical results summarized in Table 1 generally favor the conventional architecture. This outcome can be attributed to the uniform second conversion stage, which reduces the quantization error introduced in the first stage more effectively than the nonuniform second stage used in the proposed architecture when both stages operate with the same resolution (*N* = *N*_1_ = *N*_2_). The largest difference occurs at the highest considered resolution. For instance, when the total resolution *n* = 20 bits, the maximum absolute error obtained for the proposed two-stage linearizing ADC is 0.0033 °C, which is 43 times larger than the corresponding error obtained for the conventional design. For lower total resolutions, this discrepancy becomes smaller. This observation also extends to the relative nonlinearity error: for *n* = 20 bits, the proposed two-stage linearizing ADC exhibits a relative nonlinearity error of 0.0041%, which is 43 times greater than that observed in the conventional design.

Nevertheless, there are scenarios in which the proposed architecture achieves better accuracy improvement than the conventional architecture. Some of these cases are presented in Table 2. When the first and second conversion stages of the conventional architecture have different resolutions (*N*_1_ ≠ *N*_2_), while their sum remains equal to the total resolution *n* of the proposed architecture, the proposed architecture can provide higher measurement accuracy. This occurs because, although both architectures have the same total resolution, the first conversion stage in the conventional architecture, where the actual linearization takes place, operates at a lower resolution than the corresponding stage in the proposed architecture. For example, when the resolutions are *N*_1_ = 4 bits and *N*_2_ = 16 bits in the conventional architecture, the maximum absolute error amounts to 0.0919 °C, which is 28 times higher than the corresponding value obtained for the proposed architecture with the same total resolution of 20 bits, i.e., when *N* = 10 bits. This observation also applies to the relative nonlinearity error. These findings highlight the fundamental trade-off between quantization noise reduction and nonlinearity compensation. The proposed architecture shifts the balance toward nonlinearity compensation, thereby mitigating the dominant error source in thermistor-based measurement systems.

It should be noted that the transfer function of the nonuniform flash ADC is not an optimal piecewise-linear approximation of the inverse of the nonlinear input signal. Consequently, the approximation error is not fully minimized and contributes to the residual error after linearization. This observation points to a promising direction for future work, where further optimization of the nonuniform flash ADC transfer function, specifically, the selection of voltage thresholds, could lead to additional reductions in both approximation and quantization errors.

Considering that the thermistor datasheet specifies a measurement accuracy of ±1.0 °C over the considered temperature range [25], the proposed 20-bit two-stage linearizing ADC achieves a dramatic improvement, reducing the error by a factor of 303, i.e., to the value of 0.0033 °C. In addition, the relative nonlinearity error of the output voltage *V*_out_(*T*) before linearization is 4.18%, which is subsequently reduced to 0.0041% through the use of the proposed 20-bit two-stage linearizing ADC. These results clearly demonstrate the effectiveness of the proposed architecture in significantly enhancing measurement accuracy beyond the intrinsic limitations of the sensor itself, outperforming other linearization approaches reported in the literature [30,38].

The principal advantage of the proposed two-stage linearizing ADC lies in its compact structure, indicating a high potential for reduced power consumption, which resulted from the significantly reduced number of required comparators and resistors compared to the conventional architecture of the same total resolution. This reduction stems from the use of the same compact nonuniform flash ADC in both conversion stages. Consequently, both stages exhibit nonuniform characteristics, in contrast to the conventional architecture where the first stage is nonuniform and the second stage is uniform, performed by two separate flash ADCs. A quantitative comparison of the two architectures in terms of comparator and resistor counts is presented in Table 3. The corresponding expressions for the number of comparators and resistors required by the conventional (*n*_cc_ and *n*_rc_) and the proposed (*n*_cp_ and *n*_rp_) architecture, assuming identical resolutions for the first and second conversion stages (*N* = *N*_1_ = *N*_2_), are given below:

(23) $$n_{\mathrm{cc}}=2\cdot \left(2^{N}-1\right)=2^{N+1}-2,$$

(24) $$n_{\mathrm{rc}}=2\cdot 2^{N}=2^{N+1},$$

for the conventional architecture, and for the proposed architecture,

(25) $$n_{\mathrm{cp}}=2^{N-1},$$

(26) $$n_{\mathrm{rp}}=2^{N}.$$

In order to quantify the improvements achieved by the proposed compact design with respect to the conventional design, the relative differences in the numbers of required comparators and resistors, denoted by *δ*_c_ and *δ*_r_, respectively, are defined as follows:

(27) $$\delta_{c}=\frac{\left(n_{\mathrm{cc}}-n_{\mathrm{cp}}\right)}{n_{\mathrm{cc}}}\cdot 100\%,$$

(28) $$\delta_{r}=\frac{\left(n_{\mathrm{rc}}-n_{\mathrm{rp}}\right)}{n_{\mathrm{rc}}}\cdot 100\%$$

For instance, a 10-bit nonuniform flash ADC implemented according to the proposed compact design, which corresponds to a 20-bit two-stage linearizing ADC, requires a total of only 512 comparators. In contrast, a conventional two-stage linearizing ADC with the same total resolution and identical resolutions of the first and second conversion stages (*N*_1_ = *N*_2_ = 10 bits) requires 2046 comparators, which is nearly four times higher. In addition, the number of resistors employed in the conventional architecture is exactly twice the number required by the proposed architecture.

These results clearly demonstrate that the proposed architecture significantly mitigates the well-known disadvantages of flash ADCs, such as high circuit complexity and elevated power consumption, while preserving their principal advantage, extremely short conversion time resulting from the inherently parallel comparator structure.

As the total resolution *n* increases, the difference in hardware requirements between the conventional and the proposed architecture becomes even more pronounced. As previously indicated, Table 3 summarizes the total numbers of comparators and resistors required by the conventional architecture and the corresponding values for the proposed compact architecture across a range of total resolutions *n*. The table also includes the calculated relative differences in comparator counts *δ*_c_ and resistor counts *δ*_r_ between the two architectures.

The numerical results reveal a substantial reduction in hardware complexity achieved by the proposed design. With increasing resolution *n*, the relative difference in comparator counts *δ*_c_ exhibits a steady growth. The relative difference in resistor counts *δ*_r_ remains constant across all resolution values and equals 50%. For resolutions higher than *n* = 10 bits, *δ*_c_ increases rapidly, indicating that the advantages of the proposed architecture become progressively more significant at higher resolutions. The maximum relative differences will be reached at a total resolution of 38 bits, i.e., *δ*_c_ = 75% and *δ*_r_ = 50%. Nevertheless, even at relatively low resolutions, the reduction in component count remains considerable.

Such a decrease in the number of required components directly translates into several important practical benefits, including reduced silicon area, lower manufacturing cost, improved circuit compactness, and decreased power consumption. Consequently, the proposed two-stage linearizing ADC represents a particularly attractive solution for sensor linearization in embedded systems and other applications with strict constraints on power consumption, circuit complexity, and physical size.

It should also be noted that comparator C_1_ alone contributes two bits to the total resolution *n*, thereby improving the compactness of both the nonuniform flash ADC and the proposed architecture. While the comparator C_1_ and additional multiplexers introduce further propagation delay paths within the circuit, this design choice is justified by the simultaneous execution of both the linearization process and the analog-to-digital conversion. Consequently, the overall signal-processing time of the measurement system is reduced at the system level, while the implementation complexity and associated costs remain lower than those of conventional measurement systems.

Furthermore, when the resolutions of the first and second conversion stages in the conventional architecture are different (*N*_1_ ≠ *N*_2_), the hardware savings achieved by the proposed architecture become even more pronounced. This behavior is illustrated in Table 4. For example, when the conventional architecture operates with *N*_1_ = 4 bits in the first stage and *N*_2_ = 16 bits in the second stage, the relative reductions in the numbers of comparators and resistors reach 99.2189% and 98.4379%, respectively, when compared with the proposed architecture employing a single compact flash ADC with a 10-bit resolution.

Overall, these results confirm that the proposed architecture provides a highly efficient trade-off between hardware complexity, power consumption, and conversion speed. By drastically reducing the number of required comparators and resistors while maintaining high conversion speed and effective sensor linearization capability, the proposed two-stage linearizing ADC represents a compact alternative to conventional architecture, particularly in high-resolution sensing applications.

In summary, the proposed architecture should be viewed as a well-balanced trade-off between linearization accuracy and hardware efficiency, where a slight relaxation of optimal linearization performance is accepted in exchange for a significant reduction in hardware complexity, while still maintaining a high level of accuracy suitable for practical applications. Although the proposed solution may not achieve the same level of quantization noise suppression as the conventional architecture when both designs operate with the same resolutions in both stages (*N* = *N*_1_ = *N*_2_), its performance remains sufficiently high for practical temperature measurement applications. In fact, when compared with other commonly used linearization techniques for NTC thermistors, the proposed approach provides competitive accuracy while maintaining a significantly simpler hardware structure. As a result, the proposed architecture represents a highly attractive solution for practical sensor linearization applications, especially in compact and low-power measurement systems.

### Future Research Directions

Although the proposed two-stage linearizing ADC offers significant advantages in terms of compactness and cost efficiency, several aspects of its design should be considered to provide a balanced assessment of its performance.

One of the key design elements is the determination of the voltage thresholds that define the piecewise-linear segments in the first conversion stage. The procedure used in this study is intentionally simple and straightforward, with nonuniform voltage thresholds determined directly from the nonlinear input signal using a deterministic method. While this approach enables efficient hardware implementation without the need for additional digital processing or external computational resources, it introduces a residual approximation error that contributes to the overall measurement error, reflecting the underlying trade-off between implementation simplicity and approximation optimality. Nevertheless, even without explicitly minimizing the approximation error, the results demonstrate that the overall measurement error remains within acceptable limits for practical applications, thereby supporting the adequacy of the chosen approach.

The main factor that limits the ultimate measurement accuracy of the proposed architecture is quantization noise. Because the linearization is performed during the first stage of conversion, the quantization error introduced at this stage contributes to the residual system error. Although the second conversion stage refines the signal and reduces this error, a certain amount of quantization noise remains present in the final output. This effect is particularly noticeable when the resolutions of the two conversion stages are similar, since the second stage then has a limited ability to further reduce the quantization noise produced by the first stage.

Future work may focus on reducing the residual approximation error through optimized placement of voltage thresholds and on mitigating the impact of quantization noise. Additionally, combining the proposed hardware-based linearization approach with lightweight digital post-processing techniques could further improve measurement accuracy without significantly increasing system complexity. Extending the analysis to include non-ideal effects such as mismatch, noise, and offset, alongside a detailed timing overhead analysis, would represent another important direction for future research. Experimental validation through hardware implementation and testing with real sensors would also provide valuable insight into the practical performance of the proposed architecture and confirm its applicability in a wider range of sensing applications.

## 4. Conclusions

This paper presented a compact two-stage linearizing ADC intended for efficient compensation of NTC thermistor nonlinearity directly during the analog-to-digital conversion process. The proposed approach integrates the linearization mechanism into the conversion procedure itself by employing a nonuniform first conversion stage whose transfer function approximates the inverse function of the nonlinear input signal, thereby eliminating the need for additional digital post-processing and enabling a simple and efficient hardware implementation.

The operating principle of the proposed architecture was analyzed and evaluated through simulations of a complete measurement system incorporating the Vishay NTCLE428E3103F400L thermistor and its associated interface circuit. The obtained results demonstrate that the proposed linearizing ADC significantly improves the accuracy of temperature measurements. In particular, the simulations show that the absolute temperature measurement error can be reduced by approximately 303 times, i.e., to 0.0033 °C, when the proposed 20-bit two-stage linearizing ADC is applied. In addition to absolute error reduction, the proposed architecture achieves a substantial decrease in relative nonlinearity error. The relative nonlinearity error of the input voltage of the proposed two-stage linearizing ADC is 4.18% before linearization, and it is reduced to 0.0041% with the 20-bit two-stage linearizing ADC. This confirms that the proposed approach effectively compensates the dominant error source in thermistor-based measurement systems and enables high-accuracy temperature estimation.

A comparison with a conventional two-stage linearizing ADC of the same total resolution shows that the proposed solution maintains satisfactory linearization performance while achieving a substantial reduction in hardware complexity. Specifically, the required numbers of comparators and resistors are greatly reduced (by nearly 75% and 50%, respectively), resulting in a more compact circuit structure and improved cost efficiency. The benefits of the proposed architecture become even more pronounced at higher resolutions. For example, a 20-bit implementation requires only 512 comparators, compared to 2046 comparators in the conventional design, demonstrating a nearly fourfold reduction. In scenarios where the conventional architecture employs unequal stage resolutions, the proposed solution can achieve even greater hardware savings, with relative reductions reaching 99% in the number of required components. A more detailed analysis reveals that this reduction in hardware complexity stems from the use of a single compact nonuniform flash ADC across both conversion stages. This architectural feature represents a key distinction of the proposed approach, as it enables a significantly more efficient implementation.

An additional advantage lies in the simplicity of determining the voltage thresholds used in the first conversion stage. The thresholds are obtained using a straightforward procedure that does not require additional computational resources or processor-based optimization, which simplifies the overall system design and enables fully hardware-based implementation.

Overall, the proposed two-stage linearizing ADC provides an effective balance between measurement accuracy, hardware complexity, and power consumption. By combining satisfactory linearization accuracy with significantly reduced hardware requirements and enhanced potential for lower power consumption, the proposed architecture represents a practical and attractive solution for NTC thermistor linearization, particularly in compact and resource-constrained measurement systems.

## Figures and Tables

**Table 1:** Error metrics of the proposed and conventional architectures as a function of total resolution *n*. For the proposed architecture, *N* is the resolution of each stage, with (Δ*T*_max_)_p_ and (*δ*_NL_)_p_ representing the maximum absolute and relative nonlinearity errors, respectively. For the conventional architecture, *N*_1_ and *N*_2_ are the first- and second-stage resolutions, with (Δ*T*_max_)_c_ and (*δ*_NL_)_c_ representing the maximum absolute and relative nonlinearity errors, respectively.

*n* [bits]	*N*, *N*_1_, *N*_2_ [bits]	(Δ*T*_max_)_p_ [°C]	(*δ*_NL_)_p_ [%]	(Δ*T*_max_)_c_ [°C]	(*δ*_NL_)_c_ [%]
6	3	1.7404	2.1755	1.25	1.5625
8	4	0.5081	0.6351	0.3125	0.3906
10	5	0.1615	0.2019	0.0781	0.0977
12	6	0.0578	0.0722	0.0195	0.0244
14	7	0.0267	0.0334	0.0049	0.0061
16	8	0.0132	0.0165	0.0012	0.0015
18	9	0.0066	0.0082	0.0003	0.0004
**20**	**10**	**0.0033**	**0.0041**	**7.6294 × 10^−5^**	**9.5367 × 10^−5^**

**Table 2:** Error metrics of the conventional and proposed architectures as a function of resolutions *N*_1_, *N*_2_ and *N*. For the conventional architecture, *N*_1_ and *N*_2_ are the first- and second-stage resolutions, with (Δ*T*_max_)_c_ and (*δ*_NL_)_c_ representing the maximum absolute and relative nonlinearity errors, respectively. For the proposed architecture, *N* is the resolution of each stage, with (Δ*T*_max_)_p_ and (*δ*_NL_)_p_ representing the maximum absolute and relative nonlinearity errors, respectively.

Conventional Two-Stage Linearizing ADC				Proposed Two-Stage Linearizing ADC			
*N*_1_ [bits]	*N*_2_ [bits]	(Δ*T*_max_)_c_ [°C]	(*δ*_NL_)_c_ [%]	*N* [bits]	*N* [bits]	(Δ*T*_max_)_p_ [°C]	(*δ*_NL_)_p_ [%]
4	8	0.1015	0.1269	6	6	0.0578	0.0722
4	12	0.0924	0.1156	8	8	0.0132	0.0165
**4**	**16**	**0.0919**	**0.1148**	**10**	**10**	**0.0033**	**0.0041**
6	14	0.006	0.0076				

**Table 3:** Comparison of hardware complexity in terms of comparator and resistor counts for conventional and proposed architectures. The total resolution is denoted by *n*, and the resolution of each conversion stage for both architectures is *N*. The numbers of required comparators and resistors for the conventional architecture are *n*_cc_ and *n*_rc_, respectively. For the proposed architecture, the numbers of required comparators and resistors are *n*_cp_ and *n*_rp_, respectively. Finally, *δ*_c_ and *δ*_r_ represent the relative differences in the numbers of required comparators and resistors, respectively.

*n* [bits]	*N* [bits]	*n*_cc_	*n*_rc_	*n*_cp_	*n*_rp_	*δ*_c_ [%]	*δ*_r_ [%]
6	3	14	16	4	8	71.4286	50
8	4	30	32	8	16	73.3333	50
10	5	62	64	16	32	74.1935	50
12	6	126	128	32	64	74.6032	50
14	7	254	256	64	128	74.8031	50
16	8	510	512	128	256	74.902	50
18	9	1022	1024	256	512	74.9511	50
**20**	**10**	**2046**	**2048**	**512**	**1024**	**74.9756**	**50**

**Table 4:** Comparison of hardware complexity in terms of comparator and resistor counts for conventional and proposed architectures as a function of resolutions *N*_1_, *N*_2_ and *N*. For the conventional architecture, *N*_1_ and *N*_2_ are the first- and second-stage resolutions, with *n*_cc_ and *n*_rc_ representing the numbers of required comparators and resistors, respectively. For the proposed architecture, *N* is the resolution of each stage, with *n*_cp_ and *n*_rp_ representing the numbers of required comparators and resistors, respectively. Finally, *δ*_c_ and *δ*_r_ represent the relative differences in the numbers of required comparators and resistors, respectively.

Conventional Two-Stage Linearizing ADC				Proposed Two-Stage Linearizing ADC					
*N*_1_ [bits]	*N*_2_ [bits]	*n*_cc_	*n*_rc_	*N* [bits]	*N* [bits]	*n*_cp_	*n*_rp_	*δ*_c_ [%]	*δ*_r_ [%]
4	8	270	272	6	6	32	64	88.1481	76.4706
4	12	4110	4112	8	8	128	256	96.8856	93.7743
**4**	**16**	**65,550**	**65,552**	**10**	**10**	**512**	**1024**	**99.2189**	**98.4379**
6	14	16,446	16,448					96.8868	93.7743

## Data Availability

The original contributions presented in this study are included in the article. Further reasonable inquiries can be directed to the corresponding author.
